# Evaluation of the diagnostic accuracy of laboratory-based screening for hepatitis C in dried blood spot samples: A systematic review and meta-analysis

**DOI:** 10.1038/s41598-019-41139-8

**Published:** 2019-05-13

**Authors:** Sonia Vázquez-Morón, Beatriz Ardizone Jiménez, María A. Jiménez-Sousa, José M. Bellón, Pablo Ryan, Salvador Resino

**Affiliations:** 1 Unidad de Infección Viral e Inmunidad. Centro Nacional de Microbiología - Instituto de Salud Carlos III, Majadahonda, Spain; 2 Hospital General Universitario Gregorio Marañón, Madrid, Spain; 3 Instituto de Investigación Sanitaria Gregorio Marañón (IiSGM), Madrid, Spain; 4 Hospital Universitario Infanta Leonor (HUIL). Vallecas, Madrid, Spain

**Keywords:** Hepatitis, Viral infection

## Abstract

The dried blood spot (DBS) is increasingly used for the hepatitis C virus (HCV) screening. Our objective was to perform a meta-analysis of the methodology for HCV screening in DBS samples, particularly in the type of diagnostic assay used. We performed a meta-analysis of all eligible studies published to date (March 2018). The literature search revealed 26 studies: 21 for detection of anti-HCV antibodies and 10 for detection of HCV-RNA. Statistical analyses were performed using Meta-DiSc and STATA (MIDAS module). For detection of HCV antibodies, pooled diagnostic accuracy measures were as follows: sensitivity 96.1%, specificity 99.2%, positive likelihood ratio (PLR) 105, negative likelihood ratio (NLR) 0.04, diagnostic odds ratio (DOR) 2692.9, and summary receiver operating characteristic (SROC) 0.997 ± 0.001. For detection of HCV-RNA, the pooled diagnostic accuracy measures were as follows: sensitivity 97.8%, specificity 99.2%, PLR 44.8, NLR 0.04, DOR 1966.9, and SROC 0.996 ± 0.013. Similar values of pooled diagnostic accuracy measures were found according to the type of anti-HCV antibody detection assay (enzyme-linked immunosorbent assay, rapid diagnostic test, and chemiluminescence assays) and HCV-RNA detection assay (real-time polymerase chain reaction and transcription-mediated amplification). The analysis of external validity showed a high negative predicted value (NPV) for both approaches, but a low positive predicted value (PPV) when prevalence was < 10%, particularly in HCV-RNA tests. Finally, this meta-analysis is subject to limitations, especially publication bias and significant heterogeneity between studies. In conclusion, HCV screening in DBS samples has an outstanding diagnostic performance, with no relevant differences between the techniques used. However, external validity may be limited when the HCV prevalence is low.

## Introduction

About 71 million people have chronic Hepatitis C virus (HCV) infection and around 80% are undiagnosed^1^, thus leading to the development of liver disease and/or transmission of HCV infection to others unknowingly^2–4^. Furthermore, HCV diagnosis remains problematic for persons from low- and middle -income countries (LMICs) and difficult-to-access populations in developed countries (people who inject drugs [PWID], homeless people, immigrants, and sex workers), where very few individuals have access to diagnosis^2,5,6^.

The standard HCV diagnosis requires an initial serological test, followed by a confirmatory nucleic acid test (NAT) for the detection of HCV ribonucleic acid (RNA) in serum/plasma samples obtained during routine venous blood collection^4,6,7^. The tests require high-cost facilities and equipment (not always available in resource-limited settings) and specialized personnel and adequate infrastructures for the collection, transport, and storage of venous blood samples^6^. In addition, some patients, such as PWIDs, may have limited venous access, which further hampers diagnosis^2^.

Several strategies have been proposed to overcome some of these limitations^2,5,8^. One is the use of dried blood spots (DBS), which are obtained by finger puncture and depositing the blood drops on a filter paper. Such an approach can be used for HCV diagnosis in serological tests (anti-HCV antibodies) and in virological tests (HCV-RNA)^9^. DBS facilitates the sampling process by avoiding venipuncture and removing the need to separate plasma samples. In addition, DBS samples are highly stable at room temperature, and it is not necessary to maintain the cold chain for the storage of the samples and transport to the processing laboratory^10^. These advantages have made DBS sampling a promising approach to HCV screening and epidemiological surveillance in LMICs and risk groups^11–14^.

DBS is increasingly used for HCV screening, although the sensitivity and specificity of this approach for hepatitis C remain uncertain. Additionally, the laboratory methodology used for the analysis of DBS samples is very diverse, covering a wide range of settings^15–17^. In recent years, several systematic reviews and meta-analyses on this topic have been published^15–19^. However, to our knowledge, none has performed a detailed analysis of the diagnostic accuracy of laboratory-based screening for HCV. Therefore, our aim was to carefully analyze the diagnostic performance of the methodology that enables the detection of HCV infection in DBS samples, particularly in the type of diagnostic assay used, by conducting a meta-analysis of all eligible studies published to date (March 2018).

## Material and Methods

The meta-analysis was conducted following guidelines on systematic reviews and meta-analyses (PRISMA; see Supplemental File (SF) 1)^20^.

### Search strategy

Relevant studies were identified by a literature search in PubMed, Scopus, Embase, Lilacs, Web of Science, and the Cochrane Library with the following terms: (“hepatitis C” OR HCV) AND (DBS OR Whatman OR “filter paper” OR “Dried blood spot” OR “Dried blood filter” OR “Dried blood” OR “Dried sample”) AND (sensitivity OR specificity OR “Positive Predictive Value” OR “Negative Predictive Value” OR AUROC OR AUCROC OR diagnostic OR screening) NOT (review). We also reviewed the reference lists of several previously published reviews on HCV screening in DBS. The information contained in this report is based on articles published before March 2018.

### Study selection

The inclusion and exclusion criteria were established before proceeding to the search and review. The inclusion criteria were as follows: (1) studies had to have evaluated the detection of anti-HCV antibodies and/or HCV-RNA in DBS; (2) results for DBS had to have been compared with a reference method (gold standard) using serum, plasma, or whole blood samples; (3) there had to be enough available data to construct 2 × 2 tables and to calculate the number of true positives (TP), true negatives (TN), false positives (FP), and false negatives (FN). The articles excluded were as follows: (1) studies whose objective differed from that of the meta-analysis; (2) editorial comments, reviews, opinion letters, and conference proceedings; (3) studies with insufficient data to estimate the sensitivity and/or specificity of the techniques evaluated; and (4) studies in which all samples were not tested using at least a reference test or in which a reference test was performed only on a subset of samples (only positive, negative, or discordant results).

We based our selection of eligible articles on careful screening of the title and abstract; when an article fulfilled the inclusion criteria, the full text was examined, and data were extracted. Moreover, when a study included different subgroups with various diagnostic tests, only those subgroups that met the inclusion criteria were included in our meta-analysis. In the case of antibody detection assays, if a receiver operating characteristic (ROC) curve analysis was performed to determine the optimal cut-off point, we only included the data obtained with the cut-off point established by the manufacturer for the use of serum or plasma samples. However, some investigators also determined others cut-off points based on ROC curve analysis, which were excluded from the meta-analysis.

### Data extraction

Data were extracted independently by 2 investigators (B.A. and S.V.M.) and then cross-checked. When data were unclear or required assumptions to be made, other investigators (S.R. and M.A.J.S.) were consulted to reach a consensus. The values of TP, TN, FP, and FN corresponding to the tests evaluated in each article were extracted in order to create 2×2 contingency tables and calculate sensitivity and specificity. When this information was not explicitly reported, we contacted the corresponding author to request the data. If we did not receive the necessary data, the study was excluded.

### Quality assessment

Study quality and risk of bias were evaluated using QUADAS-2 (Quality Assessment of Diagnostic Accuracy studies 2)^21^, which is designed to evaluate the quality of primary diagnostic accuracy studies through 4 key domains (patient selection, index test, reference standard, and flow and timing). Each domain was assessed in terms of risk of bias (low, high, or unclear) and, in the first 3 domains, concerns about applicability (low, high, or unclear) were also considered. Two reviewers (B.A. and S.V.M.) independently assessed the study characteristics and methodological quality.

### Statistical analysis

Data were analyzed using Meta-DiSc 1.4^22^ and STATA (version 14, STATA Corp., Texas USA) with the MIDAS module. Analyses were performed according to the HCV detection method. First, we performed the analysis of HCV-antibody detection studies and HCV-RNA detection studies separately. Next, we performed a subgroup analysis within each of the above categories: anti-HCV antibody detection (enzyme-linked immunosorbent assay [ELISA], rapid diagnostic tests (RDTs), and chemiluminescence assays [CLA]) and HCV-RNA detection (real-time polymerase chain reaction [PCR] and transcription-mediated amplification [TMA]). Analyses were performed only when 3 or more articles were available.

We calculated the pooled estimates of sensitivity, specificity, positive predicted value (PPV), negative predicted value (NPV), positive likelihood ratio (PLR), negative likelihood ratio (NLR), diagnostic odds ratio (DOR), and their corresponding 95% confidence intervals (95%CI) using the accuracy data (TP, FP, FN, and TN) extracted from each eligible study. We used a random effects model (DerSimonian-Laird method), since there was significant between-study variability^23^. In order to facilitate analysis, a value of 0.5 was added to those cells that contained the value zero.

The data were displayed graphically on forest-plots, summary ROC (SROC) curves, and likelihood ratio scatter plots. Heterogeneity was measured using the Cochran’s Q test and the inconsistency index (I^2^). The Cochran’s Q was calculated as the weighted sum of squared differences between individual study effects and the pooled effect across studies and P-values were calculated by comparing the statistic with a χ2 distribution with k-1 degrees of freedom (where k is the number of studies). The I² statistic describes the percentage of total variation across studies due to heterogeneity rather than sampling error (chance). A p-value ≤ 0.05 and an I^2^ value ≥ 50% indicated significant heterogeneity. Sensitivity analyses were performed by sequential omission of individual outlier studies to investigate the influence of each study on the pooled estimates. In addition, we performed a meta-regression analysis for anti-HCV antibody detection assays and HCV-RNA detection assays with the aim of defining the potential effect of different covariates on diagnostic accuracy measures. For this purpose, we used the restricted maximum likelihood method and weighted least-squares model, with weights obtained from the study sizes. The covariates analyzed were as follows: study performed after 2010, study performed in LMICs, HIV coinfection, type of HCV detection test, capillary or venous DBS samples, and anti-HCV or HCV-RNA prevalence. A statistically significant linear relationship was one with a p-value ≤ 0.05. Publication bias was assessed using Deek’s funnel plot, and a *P* value < 0.05 indicated the presence of publication bias.

## Results

### Search results

**Figure 1:**
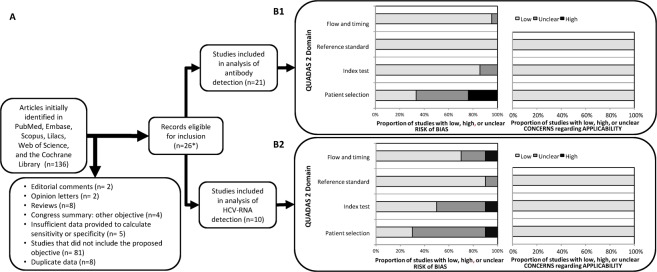
Summary of study selection process (**A**) and quality assessment using the QUADAS-2 tool (**B**): (B1) Results of quality assessment of anti-HCV antibody detection studies; (B2) Results of quality assessment of HCV-RNA detection studies. Abbreviations: HCV, hepatitis C virus; RNA, ribonucleic acid; QUADAS, quality assessment of diagnostic accuracy studies.

The literature search yielded 136 articles (Fig. 1A), of which 110 were excluded because they did not meet the inclusion criteria or presented an exclusion criterion. The remaining 26 eligible studies comprised 21 that were selected for the anti-HCV antibody detection meta-analysis^9,24–43^ and 10 for the HCV-RNA detection meta-analysis^9,24,27,38,39,44–48^. Five studies were included in both analyses^9,24,27,38,39^.

### Article characteristics

The main characteristics of the articles are summarized in Table 1 for the anti-HCV antibody detection meta-analysis and Table 2 for the HCV-RNA detection meta-analysis. The publication year of the studies ranged from 2002 to 2018. A total of 9679 DBS samples from 9679 individuals were included for anti-HCV antibody detection, and 2201 DBS samples from 2015 individuals were included for HCV-RNA detection. The median prevalence was 51.1% for anti-HCV antibodies (p25th = 24.3%; p75th = 64.3%) and 61.8% for HCV-RNA (p25th = 60.5%; p75th = 70.1%). Eleven studies provided information about HCV genotypes in infected individuals^9,24,32,36,37,40,41,45–48^. Only 7 studies included the HIV/HCV coinfection status^24,28,29,31,37,38,47^. Most of the studies (n = 15) were based on DBS samples obtained by spotting whole venous blood on filter paper, 12 were based on capillary DBS samples^24,25,27,29,31,33,37,38,41–43,49^, 2 on DBS samples obtained by either capillary or venous blood^33,41^, and 1 on mock DBS made by diluting the Second WHO International Standard for HCV-RNA in negative blood^46^.

**Table 1:** Characteristics of the Studies Included in Meta-Analysis for Antibody Detection.

Year	Author	HCV-GT	HIV status	Country	Age (years)	Males (%)	Assay in DBS samples	Gold standard	Sample (n)	HCV-infected (n)	Se (%)	Sp (%)
**ELISA**												
2013	Brandao *et al*.^25^	—	No	Brazil	40	43.8	Monolisa HCV AgAb ULTRA Assay (Bio-Rad)	Monolisa HCV AgAb ULTRA Assay (Bio-Rad)	386	40	95	100
2013	Brandao *et al*.^25^	—	No	Brazil	40	43.8	Murex HCV AgAb combination EIA (Diasorin)	Murex HCV AgAb combination EIA (DiaSorin)	386	40	82.5	98
2006	Croom *et al*.^26^	—	No	Australia	—	—	Monolisa anti-HCV PLUS Version 2 EIA (Bio-Rad)	Monolisa anti-HCV PLUS Version 2 EIA (Bio-Rad)	183	75	100	100
2014	Dokubo el al^27^	—	No	USA	< 30	—	ELISA HCV Version 3.0 (Ortho-Clinical Diagnostics)	Third-generation ELISA or CLA (Unspecified)	148	77	70.1	100
2017	Flores *et al*.^28^	—	Yes	Brazil	50.9	52	Murex anti-HCV (DiaSorin)	Murex anti-HCV (DiaSorin)	524	278	92.1	99.2
2003	Judd *et al*.^29^	—	Yes	UK	—	—	HCV 3.0 SAVe ELISA (Ortho-Clinical Diagnostics)	HCV 3.0 SAVe ELISA (Ortho-Clinical Diagnostics)	633	252	99.2	100
2013	Kania *et al*.^30^	—	No	Burkina Faso	29.8	37.2	Monolisa HCV Ab-Ag ULTRA Assay (Bio-Rad)	Monolisa HCV Ab-Ag ULTRA Assay (Bio-Rad)	218	5	100	100
2012	Larrat *et al*.^31^	—	Yes	France	46.5	61.9	Monolisa HCV Ab-Ag ULTRA Assay (Bio-Rad)	Monolisa HCV Ab-Ag ULTRA Assay (Bio-Rad)	201	113	98.2	100
2013	Lima^41^	—	No	Brazil	38.5	39.8	Murex anti-HCV v.4.0, (DiaSorin)	Murex anti-HCV v.4.0 (DiaSorin)	1043	7	57.1	100
2013	Lima^41^	—	No	Brazil	38.5	39.8	Murex anti-HCV v.4.0, (DiaSorin)	Murex anti-HCV v.4.0 (DiaSorin)	491	5	40.0	99.8
2013	Lima^41^	—	No	Brazil	38.5	39.8	Murex anti-HCV v.4.0, (DiaSorin)	Murex anti-HCV v.4.0 (DiaSorin)	254	228	91.7	92.3
2016	Marques *et al*.^32^	1b, 1a, 3	No	Brazil	47.3	43.5	HCV Ab (RADIM diagnostic)	HCV Ab (RADIM diagnostic)	99	59	94.9	100
2012	Marques *et al*.^33^	—	No	Brazil	40	43.8	HCV Ab (RADIM diagnostic)	HCV Ab (RADIM diagnostic)	411	40	97.5	99.5
2012	Marques *et al*.^33^		No	Brazil	40	43.8	ETI-AB-HCVK-4 (DiaSorin)	ETI-AB-HCVK-4 (DiaSorin)	411	45	88.9	96.1
1999	McCarron *et al*.^34^	—	No	UK			Monolisa anti-HCV (Sanofi Pasteur)	AxSYM HCV version 3.0 (Abbott Diagnostics)	220	108	100	87.5
2014	Nandagopal *et al*.^35^	—	No	India	—	—	Anti-HCV ELISA (Murex Biotech S.A)	Anti-HCV ELISA (Murex Biotech S.A)	60	31	100	100
2001	O’Brien^42^		No	USA	44.8	38	Third-generation EIA in DBS	Third-generation EIA in blood	1090	404	98.3	100
2012	Rice *et al*.^43^	—	No	UK	—	—	Chiron V3.0 SaVE assay (Ortho-Clinical Diagnostics)	Unspecified	93	59	98.3	100
2016	Soulier *et al*.^9^	1, 2, 3, 4, 5a, 6	No	France	54	55.2	aHCV VITROS ECi (Ortho-Clinical Diagnostics)	aHCV VITROS ECi (Ortho-Clinical Diagnostics)	511	341	99.1	98.2
2015	Tejada-Strop *et al*.^36^	1a, 1c, 2, 3	No	USA	—	—	HCV 3.0 EIA (Ortho-Clinical Diagnostics)	HCV 3.0 EIA (Ortho-Clinical Diagnostics)	103	52	90	100
2010	Tuaillon *et al*.^37^	1, 2, 3, 4	Yes	France	—	—	HCV 3.0 EIA (Ortho-Clinical Diagnostics)	HCV 3.0 EIA (Ortho-Clinical Diagnostics)	200	100	99	98
2018	Vázquez-Morón *et al*.^24^	1a, 1b, 2, 3, 4, 5	Yes	Spain	44.3	66.9	Murex anti-HCV kit, v.4.0 (DiaSorin,)	ADVIA Centaur® HCV assay	139	108	92.6	100
**Chemiluminescence immunoassays**												
2016	Mössner *et al*.^38^	—	Yes	Denmark	—	—	Architect anti-HCV assay (Abbott Diagnostics)	Architect anti-HCV assay (Abbott Diagnostics)	404	116	96.6	100
2013	Ross *et al*.^39^	—	No	Germany	—	—	Architect anti-HCV assay (Abbott Diagnostics)	Architect anti-HCV assay (Abbott Diagnostics)	339	179	97.8	100
2015	Tejada-Strop *et al*.^36^	1a, 1c, 2, 3	No	USA	—	—	VITROS anti-HCV IgG CLA (Ortho-Clinical Diagnostics)	VITROS anti-HCV IgG CLA (Ortho-Clinical Diagnostics)	103	52	92	100
**Rapid diagnostic tests**												
2012	Larrat *et al*.^31^	—	Yes	France	46.5	61.9	OraQuick HCV Rapid Antibody Test (OraSure Technologies)	Monolisa HCV AbAg ULTRA Assay (Bio-Rad)	201	113	97.4	100
2016	Poiteau *et al*.^40^	1a, 1b, 2, 3a, 4, 6	No	France	—	—	OraQuick HCV Rapid Antibody Test (OraSure Technologies)	aHCV VITROS ECi (Ortho-Clinical Diagnostics	207	139	100	100
2016	Poiteau *et al*.^40^	1a, 1b, 2, 3a, 4, 6	No	France	—	—	First Response® HCV Card Test (Premier Medical Corporation Ltd)	aHCV VITROS ECi (Ortho-Clinical Diagnostics	207	139	99.3	100
2016	Poiteau *et al*.^40^	1a, 1b, 2, 3a, 4, 6	No	France	—	—	Assure HCV Rapid Test (MP Diagnostics)	aHCV VITROS ECi (Ortho-Clinical Diagnostics	207	139	98.6	100
2016	Poiteau *et al*.^40^	1a, 1b, 2, 3a, 4, 6	No	France	—	—	MultiSure HCV Antibody Assay (MP Diagnostics)	aHCV VITROS ECi (Ortho-Clinical Diagnostics)	207	139	98.6	100

Abbreviations: Ab, antibody; Ag, antigen; Anti-HCV, HCV antibodies; CLA, chemiluminescence assay; DBD, dried blood spot; EIA, enzyme immunoassay; ELISA, enzyme-linked immunosorbent assay; GT, genotype; HCV, hepatitis C virus; HIV, human immunodeficiency virus; Se, sensitivity; Sp, specificity; TMA, transcription mediated amplification.

**Table 2:** Characteristics of Studies Included in the Meta-Analysis With Respect to HCV-RNA Detection.

Year	Author	HCV-GT	HIV	Country	Age (years)	Males (%)	Assay in DBS samples	Gold standard	Sample (n)	HCV-infected (n)	Se (%)	Sp (%)
**2012**	Bennett *et al*.^46^	1a, 2b, 3a, 4a, 5, 6a	No	United Kingdom	—	—	TaqMan real-time PCR (In-house 2-step RT and PCR)	Real-time RT-PCR: m2000 rt (Abbott Molecular)	80	57	100	95.8
**2010**	De Crignis *et al*.^47^	1a, 1b, 2a, 3a	Yes	Italy	—	—	SYBR Green real-time RT-PCR for HCV and HIV detection	Real-time RT-PCR: VERSANT HCV RNA 3.0 b-DNA Assay (Siemens)	25	16	93.8	100
**2014**	Dokubo *et al*.^27^	—	No	USA	< 30	—	dHCV TMA (Novartis Vaccines and Diagnostics)	TMA: Procleix Ultrio Assay (Novartis Vaccines and Diagnostics) s	132	48	89.6	100
**2016**	Mössner *et al*.^38^	—	Yes	Denmark	—	—	TMA: Procleix Ultrio Elite assay (Novartis Vaccines and Diagnostics)	TMA: Procleix Ultrio Elite Assay (Novartis Vaccines and Diagnostics)	107	85	95.3	95.5
**2013**	Ross *et al*.^39^	—	No	Germany	—	—	TMA: VERSANT HCV RNA Qualitative Assay (Siemens)	TMA: VERSANT HCV RNA Qualitative Assay (Siemens)	150	100	100	100
**2018**	Saludes *et al*.^45^	1, 2, 3, 4	No	Spain	—	—	QuantiFast Pathogen RT-PCR + IC Kit (QIAGEN)	Abbott RealTime HCV (Abbott Molecular)	82	38	100	100
**2012**	Santos *et al*.^44^		No	Brazil	—	—	In-house qPCR	In-house qPCR	168	101	98	94.3
**2002**	Solmone *et al*.^48^	1b, 2a, 2c, 3a, 4c, 4d	No	Italy	—	—	In-house 2-step RT and PCR	Amplicor HCV Monitor (Roche Molecular) TMA: VERSANT HCV RNA Qualitative Assay (Siemens)	55	34	100	100
**2002**	Solmone *et al*.^48^	1b, 2a, 2c, 3a, 4c, 4d	No	Italy	—	—	TMA Unspedified.	Amplicor HCV Monitor (Roche Molecular) TMA: VERSANT HCV RNA Qualitative Assay (Siemens)	55	34	100	100
**2016**	Soulier *et al*.^9^	1, 2, 3, 4, 5a, 6	No	France	54	55,2	Real-time RT-PCR: Cobas Ampliprep/Cobas TaqMan HCV version 2 (Roche Molecular)	Real-time RT-PCR: Cobas Ampliprep/Cobas TaqMan HCV version 2 (Roche Molecular)	511	315	97.1	100
**2016**	Soulier *et al*.^9^	1, 2, 3, 4, 5a, 6	No	France	54	55,2	Real-time RT-PCR: m2000rt (Abbott Molecular)	Real-time RT-PCR: m2000rt (Abbott Molecular)	511	314	98.1	100
**2018**	Vázquez-Morón *et al*.^24^	1a, 1b, 2, 3, 4, 5	Yes	Spain	44.3	66.9	Quantitec SYBR Green RT-PCR One Step kit (Qiagen)	VERSANT HCV RNA 1.0 Assay	139	108	99.1	100

Abbreviations: dHCV TMA, discriminatory HCV transcription-mediated amplification assay; GT, genotype; HCV, hepatitis C virus; HIV, human immunodeficiency virus; PCR, polymerase chain reaction; RT, reverse transcription; Se, sensitivity; Sp, specificity; TMA, transcription-mediated amplification.

Quality was assessed using QUADAS-2 (Fig. 1 and SF 2). Quality scores are summarized in Fig. 1B1 for the anti-HCV antibody detection assays and Fig. 1B2 for the HCV-RNA detection assays. The overall quality of the studies was moderate and most did not use a random or consecutive sampling method. Additionally, a significant percentage of studies did not hide the results of the reference test or did not report blinding of laboratory personnel to the results of the reference test. As for concerns about the applicability of the studies, the risk of bias was low in all cases.

### Meta-analysis of anti-HCV antibody detection in DBS samples

A total of 30 anti-HCV antibodies assays from 21 different studies were analyzed: 22 ELISA^9,24–37,41–43^, 5 RDT^31,40^, and 3 CLA^36,38,39^. Publication bias was detected using Deek’s funnel plot asymmetry test (*P* = 0.001) (SF 3).

**Figure 2:**
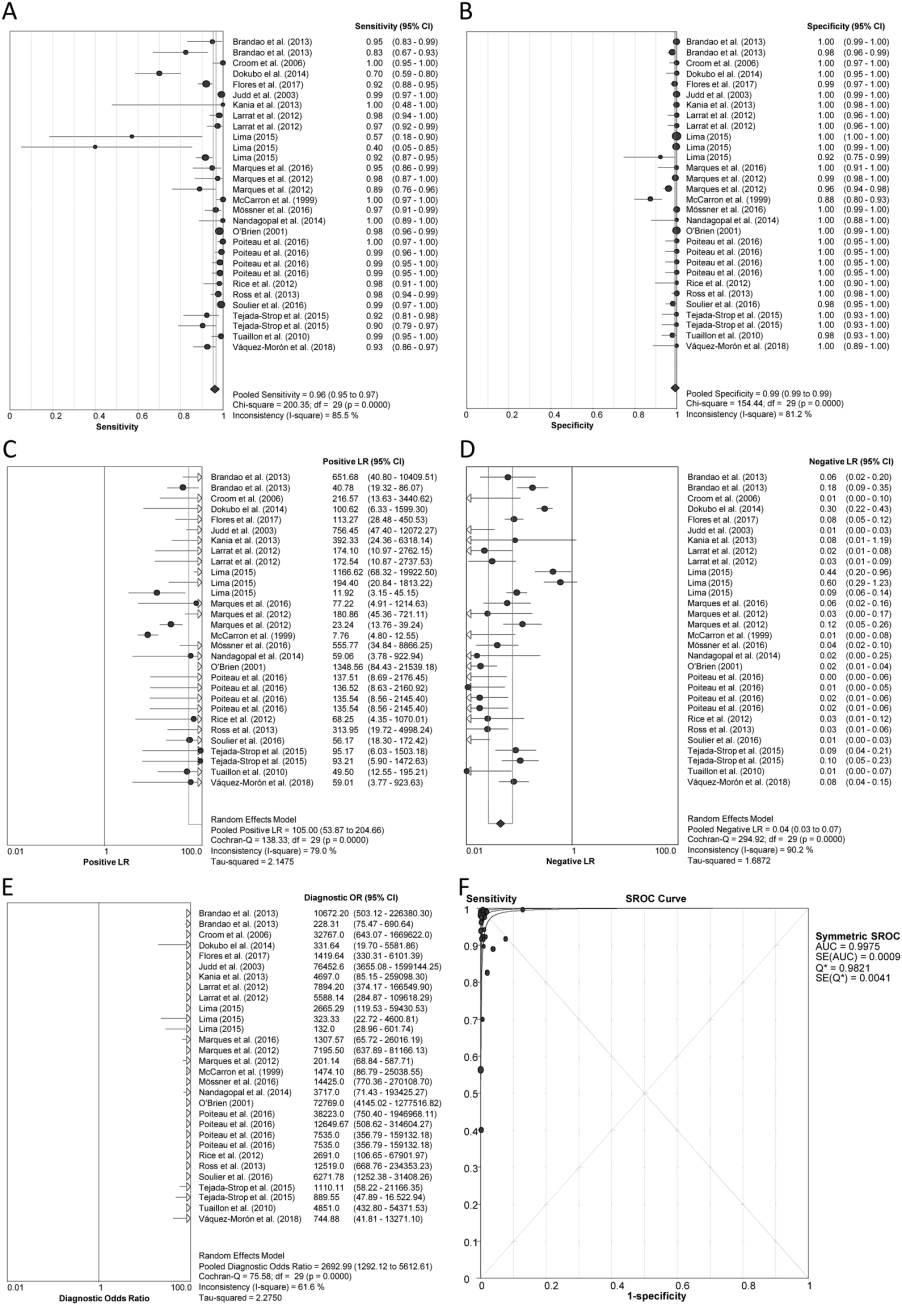
Anti-HCV antibody assays. Forest plot of sensitivity (**A**), specificity (**B**), positive LR (**C**), negative LR (**D**), diagnostic odds ratio (**E**), and SROC plot (**F**). Abbreviations: LR, likelihood ratio; SROC, summary of receiver operating characteristic; HCV, hepatitis C virus.

**Figure 3:**
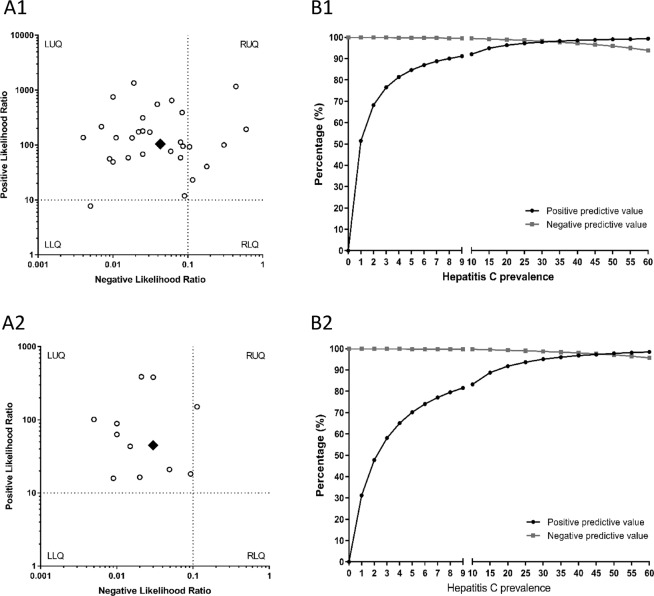
The likelihood scatter-plot (A1 & A2) and theoretical values of positive predicted value (PPV) and negative predicted value (NPV) against increasing anti-HCV antibody prevalence values (B1) and HCV-RNA prevalence (B2). Abbreviations: HCV, hepatitis C virus; RNA, ribonucleic acid; LUQ, left upper quadrant; RUQ, right upper quadrant; LUQ, left lower quadrant; LUQ, right lower quadrant.

Pooled diagnostic accuracy measures are shown in Fig. 2. Sensitivity was 96.1% (95%CI = 95.4%; 96.7%), specificity 99.2% (95%CI = 99.0%; 99.4%), PLR 105.0 (95%CI = 53.8; 204.6), NLR 0.04 (95%CI = 0.03; 0.07), DOR 2692.9 (95%CI = 1292.1; 5612.6), and SROC 0.997 ± 0.001, indicating high diagnostic accuracy. All statistical measures showed values of I^2^ > 50% (*P* < 0.05), suggesting substantial heterogeneity. However, according to the sensitivity analysis, no individual study had a significant influence on pooled estimates. The likelihood scatter-plot (Fig. 3A1) shows a matrix of values of PLR and NLR. Most studies were in the left upper quadrant (LUQ), which means that anti-HCV antibody detection tests from DBS samples are useful for confirmation and exclusion of exposure to HCV. We also calculated the theoretical values of PPV and NPV using pooled values of PLR (105.0) and NLR (0.04) and plotted them against increasing anti-HCV antibody prevalence (Fig. 3B1, full description in SF 4). We found a PPV of 84.7% for a 5% prevalence and 92.1% for a 10% prevalence, whereas NPV continued with values above 93% when prevalence reached 60%.

We also performed an analysis according to the type of anti-HCV antibody detection assay: ELISA (SF 5), RDT (SF 1), and CLA (SF 7). Diagnostic accuracy measures were similar for ELISA, RDT, and CLA, and in turn similar to the global estimates for anti-HCV antibody detection (Fig. 2). For ELISA, PPV was 82.5% for a 5% prevalence and 90.8% for a 10% prevalence; for RDT, PPV was 88.3% for a 5% prevalence and 94.1% for a 10% prevalence; and for CLA, PPV was 93.1% for a 5% prevalence and 96.6% for a 10% prevalence, whereas NPV was ≥ 91% for ELISA, RDT, and CLA when prevalence reached 60% (SF 4).

Meta-regression results are shown in SF 7. In the univariate analysis, we found significant values for studies performed after 2010 (p = 0.041) and in LMICs (p < 0.001). However, in the multivariate analysis, none of the factors had a significant potential effect on the diagnostic accuracy of the anti-HCV antibody detection assays.

### Meta-analysis of HCV-RNA detection in DBS samples

A total of 12 HCV-RNA detection assays from 10 different studies were analyzed: 9 PCR assays^9,24,39,45–49^ and 3 TMA assays^27,38,48^. Deek’s funnel plot asymmetry test revealed publication bias (*P* = 0.009) (SF 9).

**Figure 4:**
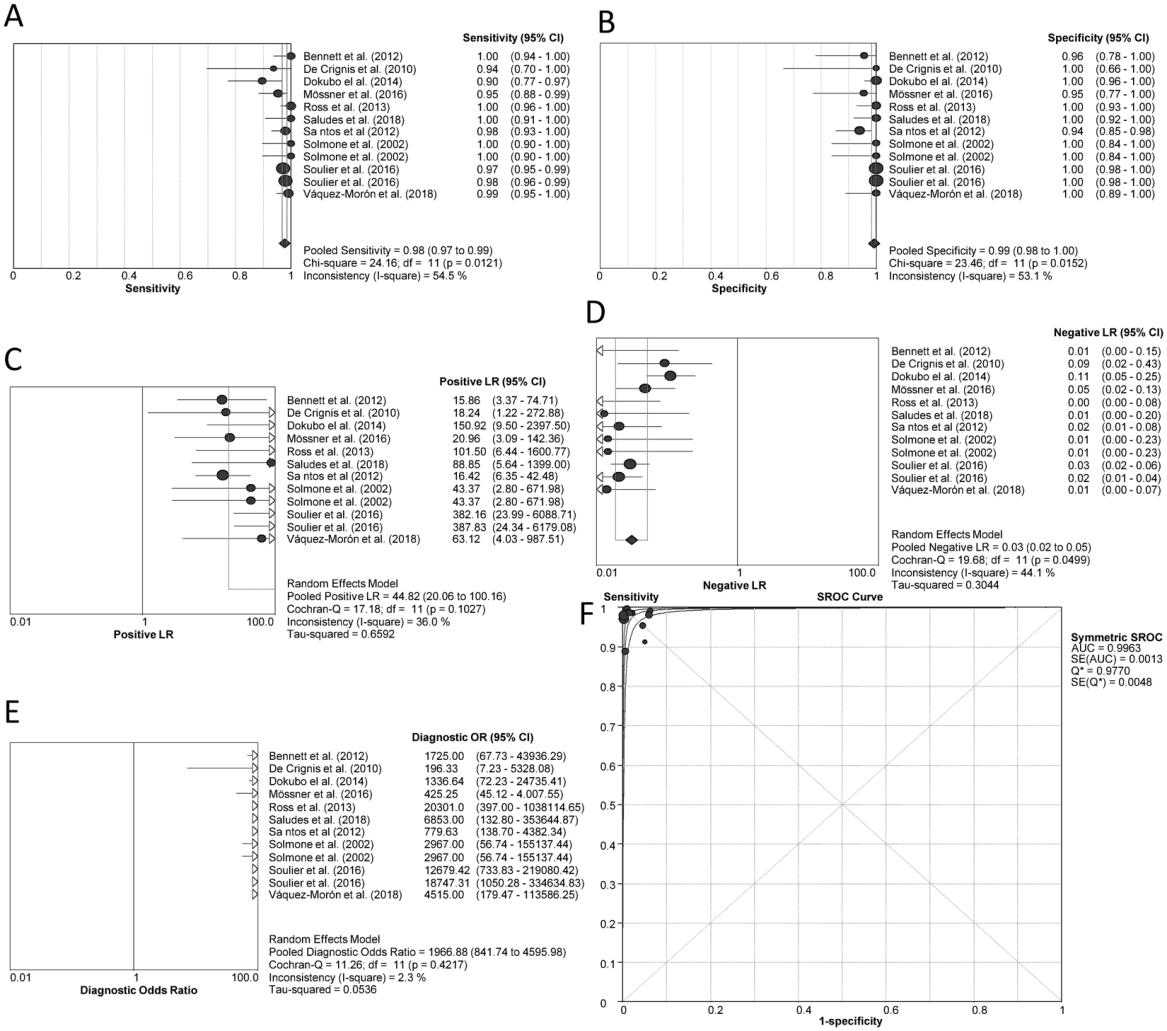
HCV-RNA detection assays. Forest plot of sensitivity (**A**), specificity (**B**), positive LR (**C**), negative LR (**D**), diagnostic odds ratio (**E**), and SROC plot (**F**). Abbreviations: LR, likelihood ratio; SROC, summary of receiver operating characteristic; HCV, hepatitis C virus; RNA, ribonucleic acid.

Pooled diagnostic accuracy measures are shown in Fig. 4. Sensitivity was 97.8% (95%CI = 96.8%; 98.5%), specificity 99.2% (95%CI = 98.3%; 99.7%), PLR 44.8 (95%CI = 20.1; 100.2), NLR 0.04 (95%CI = 0.02; 0.06), DOR 1966.9 (95%CI = 841.7; 4596.0), and SROC 0.996 ± 0.013, indicating high diagnostic accuracy. Sensitivity and specificity showed I^2^ > 50% (*P* < 0.05), suggesting considerable heterogeneity, whereas the other statistical measures had I^2^ < 50%. No individual study showed a significant influence on pooled estimates in the sensitivity analyses. The likelihood scatter-plot (Fig. 3A2) also showed that most of the studies were in the LUQ. Thus, HCV-RNA detection tests in DBS samples are also useful for confirmation and exclusion of HCV infection. The theoretical values of PPV and NPV were calculated using the pooled PLR (44.82) and NLR (0.03) values and plotted against increasing HCV-RNA prevalence values (Fig. 3B2, full description in SF 4). We found a PPV of 70.2% for a 5% prevalence and 83.3% for a 10% prevalence, whereas NPV continued with values above 95% when prevalence reached 60%.

We also performed an analysis according to the type of HCV-RNA detection assay: PCR (SF 10) and TMA (SF 11). Pooled diagnostic accuracy measures were similar between PCR and TMA, and in turn similar to global estimates for HCV-RNA detection (Fig. 4). For PCR, PPV was 72.3% for a 5% prevalence and 84.7% for a 10% prevalence; for TMA, PPV was 68.1% for a 5% prevalence and 81.9% for a 10% prevalence, whereas NPV was > 90% for PCR and TMA when prevalence reached 60% (SF 4).

Meta-regression results are shown in SF 12. We found no significant values in the univariate or the multivariate analysis. Therefore, no factors showed a significant potential effect on the diagnostic accuracy of the HCV-RNA detection assays.

## Discussion

In this meta-analysis, we found that the internal validity of HCV screening tests in DBS samples was very high: pooled sensitivity values were > 96% and specificity values were > 99%. The diagnostic performance of anti-HCV antibody tests (ELISA, RDT, and CLA) was slightly lower than that of HCV-RNA tests (PCR and TMA), with the latter having higher sensitivity values. However, SROC values were > 0.985 in all of the tests analyzed.

Likelihood ratios summarize the ability of a test to predict the probability of disease. The positive value confirms the reliability of a positive test, whereas the negative value calls a normal result into question. Additionally, the characteristics of diagnostic tests (relating to the detection or exclusion of the condition of interest) may be described in terms of likelihood scatter-plots and the threshold values used to recommend a test for clinical use, namely, 0.1 for NLR and 10.0 for PLR^50^. In our study, most of studies were in the LUQ (NLR < 0.1 and PLR > 10.0), indicating that HCV screening tests in DBS samples may be useful for either confirmation or exclusion of exposure to HCV and active infection.

We also evaluated theoretical values of PPV and NPV, which were calculated to evaluate the external validity of HCV screening, against the increasing prevalence of HCV (see SF 3). We found similar NPV values owing to the high specificity of both approaches. It should be noted that NPV values were > 99% for an HCV prevalence < 10% and did not undergo a marked decline at higher prevalence values (NPV > 90% at 60% HCV prevalence), thus indicating that HCV screening could be used in DBS samples owing to the low number of false negatives it produces. However, PPV values were not uniform in all the techniques. The PPV for anti-HCV antibody tests was higher than for HCV-RNA tests, particularly when the prevalence of HCV was lower than 10%. Furthermore, RDT and CLA assays showed higher PPV values than ELISA assays, whereas in HCV-RNA assays, PCR showed higher PPV values than TMA assays when prevalence was < 10%. When we evaluated the possible use of these tests with an HCV prevalence < 5%, we found PPV values < 90% for anti-HCV antibody tests (except CLA) and < 85% for HCV-RNA, both of which are highly undesirable for diagnostic assays in the general population of most countries (HCV prevalence < 2.5%)^51^. In contrast, at an HCV prevalence > 10%, the PPV was for anti-HCV antibody tests > 90%, which is within acceptable limits for HCV screening in DBS samples and could be used in high-prevalence populations, such as PWID, prisoners or ex-prisoners, sex workers, and MSM^3^. In these key populations, screening for and treatment of hepatitis C and other interventions that reduce HCV transmission seem to be cost-effective^52–55^. In addition, use of DBS may increase uptake of HCV testing^19^, thus enabling scale-up of HCV treatment and other interventions to minimize HCV transmission^54,55^. However, further research on the cost-effectiveness of HCV screening in DBS samples in at-risk populations is needed before its use can be generalized. Additionally, it would be applicable to the general population in countries with a high prevalence of HCV, such as Egypt (14.7%), Cameroon (13.8%), Burundi (11.3%), and Uzbekistan (11.3%), where significant iatrogenic transmission has been reported^51^. Therefore, HCV screening in DBS samples with anti-HCV antibody tests may be an ideal diagnostic assay in endemic areas with a higher HCV prevalence, especially in populations where there is limited access to health services, laboratory facilities, or rapid diagnostic tests. However, HCV-RNA tests showed PPV values < 90%, particularly TMA assays (with 81.9% when prevalence was 10%), which would not be appropriate for HCV screening as a single laboratory test. However, it should improve when using the reference two-step HCV screening algorithm based on serological screening followed by molecular confirmation of serology positives. Thus, HCV-RNA tests should be used with some reservations, depending on the technique applied and the expected HCV prevalence.

The accuracy of a systematic review depends on the quality of the studies included. The quality of the articles included in the present meta-analysis was moderate, since many items regarding the risk of bias were lacking or unclear. Some of the main drawbacks were that most of the studies were cross-sectional studies, whose design resembled that of case-control studies, thus providing little information about the reference standard and index test and unclear data on flow and timing information. Moreover, a high percentage of DBS samples were not collected at the point of care under real-world conditions. Therefore, the number of high-quality articles was moderate, and an analysis based only on high-quality studies (ie, with a low risk of bias) could not be performed.

In this meta-analysis, we evaluated the heterogeneity by the tests of Cochran’s Q and I². However, Cochran’s Q test is known to be poor at detecting true heterogeneity among studies, particularly when meta-analyses include small numbers of studies; whereas this test may have excessive power when there are many studies, particularly when those studies are large^56^. Moreover, I² test is a preferable measurement to evaluate the heterogeneity because it is easy to calculate, has values between 0 and 100 (intuitive interpretation), focuses attention on the effect of any heterogeneity on the meta-analysis, is independent of the number of studies included in the meta-analysis and the type of outcome data^56^. In our study, we found significant heterogeneity among the studies, which is a common and expected characteristic in a meta-analysis of diagnostic tests. To our knowledge, there is no real consensus on how to interpret the heterogeneity measures in meta-analysis. In the opinion of many researchers, this depends on the type of meta-analysis. When performing a meta-analysis of clinical trials that evaluate the effects of a treatment and that included studies for estimating the same outcome, the heterogeneity should be low. However, the situation is different when pooling results from epidemiological studies. In our case, we performed a meta-analysis of epidemiological studies that had different designs and objectives, and which measured also different outcomes. In this type of meta-analysis, there is no way to control all possible confounding factors, so substantial heterogeneity between the studies can be expected. In this situation, a random effects analysis should be performed on the assumption that the different studies measures different things^23^. This heterogeneity may have been due to a large series of factors that could have influenced our results, such as sample size, reference tests, screening tests, HCV prevalence, HCV viral load, HCV genotype, HIV coinfection, DBS elution method, RNA extraction method, HCV-RNA amplification technique, type of PCR used, and the region of the amplified HCV genome. Some of these factors were analyzed, although others were not owing to the lack of data in the articles included. We analyzed whether heterogeneity was due to differences between the types of tests used in each study. All pooled measures for anti-HCV antibody assays showed high heterogeneity. A subgroup analysis was then performed according to the technique used (ELISA, TDR, or CLA) to investigate potential sources of heterogeneity. Thus, the HCV screening test used seemed to be responsible for part of the heterogeneity, since RDT and CLA became homogeneous once the analysis was stratified by the type of test. However, heterogeneity was maintained in the ELISA group, probably because of the high variability in the ELISA tests used by the authors. Furthermore, HCV-RNA tests were less heterogeneous, and when the HCV-RNA techniques (PCR or TMA) were analyzed separately, we found higher heterogeneity in TMA than in PCR. On the other hand, a meta-regression analysis found that several factors had no influence on the diagnostic accuracy of HCV screening. No individual study showed significant influence on pooled estimates when sensitivity analyses were performed, thus indicating the reliability and stability of our results.

Generalizability of HCV screening in DBS samples remains limited in specific circumstances. On the one hand, no uniform protocols were applied in most of studies included in our meta-analysis. For example, the HCV screening kits were generally from different companies, and when they were from the same company, they could have been subject to changes in the manufacturing process over the years. Manufacturers should formally validate their DBS assays for use with their commercial assays. Also important is the optimal pre-analytical treatment of DBS samples in relation to storage conditions (eg, temperature, humidity) and sample elution. Manufacturers should establish protocols for optimal pre-analytical treatment of DBS specimens. Further studies are needed to demonstrate the stability of DBS under different conditions. In our meta-analysis, a significant percentage of studies (35%) did not report information about storage conditions, with the result that sample storage conditions were not included in the meta-regression. In addition, the specifications of the storage conditions were very different, thus making it difficult to create a variable that can be entered into the meta-analysis. When the articles included this information, the storage conditions were very far from real-world conditions in LMICs, because most studies were from high-income countries. Future studies on diagnostic accuracy should assess the impact of environmental conditions common to low-resource field settings.

The intrinsic validity of a diagnostic test is obtained by calculating the values of sensitivity and specificity. Therefore, the diagnostic tests analyzed in this meta-analysis were quite valid, since the sensitivity was > 96% and specificity was > 99%. Only a small margin of improvement remains to raise the sensitivity to 99–100%, which could be achieved with assays and methodologies more appropriate for the use of DBS in the HCV screening. Moreover, predictive values provide relevant information when making a clinical decision about a specific test result, but it has the limitation that they depend on the prevalence of the disease to be diagnosed in the study population. Thus, the low prevalence of HCV infection ( < 5%) affects the external validity of the tests since affects the positive and negative predictive values.

In two recent meta-analyses, Lange *et al*.^15,16^ evaluated the diagnostic accuracy of HCV screening according to the type of laboratory methodology. However, in our opinion, our meta-analysis has a series of merits that provide a differential added value. In contrast to Lange *et al*., we showed pooled diagnostic accuracy measures (sensitivity, specificity, PPV, NPV, PLR, NLR, DOR, and SROC) according to the type of laboratory assay used for HCV screening (anti-HCV antibody assays [ELISA, TDR, and CLA] and HCV-RNA tests [PCR and TMA]) in the same meta-analysis. We showed both the internal validity and the external validity (PPV and NPV against the increasing prevalence of HCV) of the techniques used. We also analyzed publication bias and the impact of covariables on effect size using regression-based techniques.

Finally, in order to ensure that our results were interpreted appropriately, a series of points had to be taken into account. Firstly, this meta-analysis revealed a publication bias, indicating that there was a higher tendency to publish studies with favorable results than studies with unfavorable results. Secondly, the number of studies in some subgroup analyses was small, thus potentially leading to weak results. Thirdly, the fact that most of the studies included were conducted in Western countries and South America does not prevent DBS samples from being used in other parts of the world (eg, Asia), provided similar technology and protocols are applied. Fourthly, we have not done a study of cost-effectiveness, which is an important consideration in most public health interventions, especially in LMICs.

In the current era of direct-acting antivirals for HCV treatment, there is a growing need for rapid, sensitive, and specific identification of HCV-infected individuals to enable effective prompt HCV therapy, implement HCV-specific infection control measures, and decrease HCV prevalence. HCV screening tests in DBS samples may have the potential to meet some of these needs, although their current limitations in diagnostic performance should be considered. More sensitive assays must be developed to increase the potential of DBS as an HCV screening tool.

## Conclusions

In conclusion, HCV screening from DBS samples has an outstanding diagnostic performance, with no relevant differences in diagnostic accuracy according to the type of technique used for detection of anti-HCV antibodies or HCV-RNA. However, external validity may be limited when the prevalence of HCV is low. This approach could be useful for the implementation of national programs for the control of HCV infections in LMICs and in difficult-to-access populations. Further studies to determine diagnostic accuracy in real-world settings are needed.

## Supplementary information

Supplemental File 1

Supplemental File 2

Supplemental File 3

Supplemental File 4

Supplemental File 5

Supplemental File 6

Supplemental File 7

Supplemental File 8

Supplemental File 9

Supplemental File 10

Supplemental File 11

Supplemental File 12

## Data Availability

All relevant data are within the paper and its Supporting Information files.
