# Supplementary material for: Evaluation of the diagnostic accuracy of laboratory-based screening for hepatitis C in dried blood spot samples: A systematic review and meta-analysis
Source: Sci Rep. 2019 May 13;9:7316. doi: 10.1038/s41598-019-41139-8 (PMC6514168; doi:10.1038/s41598-019-41139-8)
Supplement: Supplementary file 2 — Supplemental File 2 [file 41598_2019_41139_MOESM2_ESM.pdf]

TITLE PAGE

Title: Evaluation of the diagnostic accuracy of laboratory-based screening for hepatitis C in dried blood spot samples: A systematic review and meta-analysis

Running head: HCV screening in DBS samples

Authors: Sonia VÁZQUEZ-MORÓN [1(¥)]; Beatriz ARDIZONE JIMÉNEZ [1(¥)]; María A. JIMENEZ-SOUSA [1]; José M BELLON [2,3]; Pablo RYAN [4]; Salvador RESINO [1(*)]

(¥), Both authors contributed equally to this study; (*), Corresponding author

Current affiliations: (1) Unidad de Infección Viral e Inmunidad. Centro Nacional de Microbiología - Instituto de Salud Carlos III, Majadahonda, Spain; (2) Hospital General Universitario Gregorio Marañón, Madrid, Spain; (3) Instituto de Investigación Sanitaria Gregorio Marañón (IiSGM), Madrid, Spain; (4) Hospital Universitario Infanta Leonor (HUIL). Vallecas, Madrid, Spain.

Corresponding authors: Salvador Resino, Centro Nacional de Microbiología, Instituto de Salud Carlos III (Campus Majadahonda); Carretera Majadahonda- Pozuelo, Km 2.2; 28220 Majadahonda (Madrid), Spain. Tel: +34 918 223 266; Fax: +34 915 097 946; e-mail: sresino@isciii.es

Declarations of interest: none.

Author contributions:

Sonia Vázquez-Morón: investigation, methodology, writing – original draft

Beatriz Ardizone: investigation, methodology, writing – original draft

María A Jiménez-Sousa: investigation, methodology, writing – review and editing

José M Bellón: methodology: statistical analysis

Pablo Ryan: writing – review and editing

Salvador Resino: conceptualization, formal analysis, writing – original draft, supervision

Character count of Title: 150

Character count of Running Head: 28

Word count of Abstract: 257

Word count of Keywords: 5

Words count for main body: 4476

Count of References: 56

Count of Tables: 2

Count of Figures: 4

Count of Suppl. Data: 12

Supplemental File 2. Summary of the quality assessment by using QUADAS-2.

Bias (Anti – HCV Ab)

	PATIENT SELECTION	INDEX TEST	REFERENCE STANDARD	FLOW AND TIMING
Brandao et al (2013)	Low	Low	Low	Low
Croom et al (2006)	Low	Low	Low	Low
Dokubo el al (2014)	Low	Unclear	Low	Low
Flores et al (2017)	Unclear	Low	Low	Low
Judd et al (2003)	Unclear	Low	Low	Unclear
Kania et al (2013)	Low	Low	Low	Low
Larrat et al (2012)	Low	Low	Low	Low
Lima (2015)	Low	Low	Low	Low
Marques et al (2016)	Low	Low	Low	Low
Marques et al (2012)	Low	Low	Low	Low
Mc Carron et al (1999)	Low	Unclear	Low	Low
Mössner et al (2016)	Low	Low	Low	Low
Nandagopal et al (2014)	Low	Low	Low	Low
O'Brien et al (2001)	Low	Low	Low	Low
Poiteau et al (2015)	Low	Unclear	Low	Low
Rice et al (2012)	Low	Low	Low	Low
Ross et al (2013)	Low	Low	Low	Low
Soulier et al (2016)	Low	Low	Low	Low
Tejada-Strop et al (2015)	Low	Low	Low	Low
Tuaillon et al (2010)	Low	Low	Low	Low
Vázquez-Morón et al (2018)	Low	Low	Low	Low

Applicability (Anti – HCV Ab)

	PATIENT SELECTION	INDEX TEST	REFERENCE STANDARD
Brandao et al (2013)	Low	Low	Low
Croom et al (2006)	Low	Low	Low
Dokubo el al (2014)	Low	Low	Low
Flores et al (2017)	Low	Low	Low
Judd et al (2003)	Low	Low	Low
Kania et al (2013)	Low	Low	Low
Larrat et al (2012)	Low	Low	Low
Lima (2015)	Low	Low	Low
Marques et al (2016)	Low	Low	Low
Marques et al (2012)	Low	Low	Low
Mc Carron et al (1999)	Low	Low	Low
Mössner et al (2016)	Low	Low	Low
Nandagopal et al (2014)	Low	Low	Low
O'Brien et al (2001)	Low	Low	Low
Poiteau et al (2015)	Low	Low	Low
Rice et al (2012)	Low	Low	Low
Ross et al (2013)	Low	Low	Low
Soulier et al (2016)	Low	Low	Low
Tejada-Strop et al (2015)	Low	Low	Low
Tuaillon et al (2010)	Low	Low	Low
Vázquez-Morón et al (2018)	Low	Low	Low

Bias (NAT)

	PATIENT SELECTION	INDEX TEST	REFERENCE STANDARD	FLOW AND TIMING
Bennett et al (2012)	Low	High	Low	Low
De Crignis et al (2010)	Low	Unclear	Low	Low
Dokubo el al (2014)	Low	Unclear	Low	Low
Mössner et al (2016)	Low	Low	Low	High
Ross et al (2013)	Low	Low	Low	Unclear
Saludes et al (2018)	Low	Unclear	Low	Low
Santos et al (2012)	Low	Low	Low	Low
Solmone et al (2002)	Unclear	Unclear	Unclear	Unclear
Soulier et al (2016)	Low	Low	Low	Low
Vázquez-Morón et al (2018)	Low	Low	Low	Low

Applicability (NAT)

	PATIENT SELECTION	INDEX TEST	REFERENCE STANDARD
Bennett et al (2012)	Low	Low	Low
De Crignis et al (2010)	Low	Low	Low
Dokubo el al (2014)	Low	Low	Low
Mössner et al (2016)	Low	Low	Low
Ross et al (2013)	Low	Low	Low
Saludes et al (2018)	Low	Low	Low
Santos et al (2012)	Low	Low	Low
Solmone et al (2002)	Low	Low	Low
Soulier et al (2016)	Low	Low	Low
Vázquez-Morón et al (2018)	Low	Low	Low
