# Supplementary material for: Evaluation of the diagnostic accuracy of laboratory-based screening for hepatitis C in dried blood spot samples: A systematic review and meta-analysis
Source: Sci Rep. 2019 May 13;9:7316. doi: 10.1038/s41598-019-41139-8 (PMC6514168; doi:10.1038/s41598-019-41139-8)

TITLE PAGE

Title: Evaluation of the diagnostic accuracy of laboratory-based screening for hepatitis C in dried blood spot samples: A systematic review and meta-analysis

Running head: HCV screening in DBS samples

Authors: Sonia VÁZQUEZ-MORÓN [1(¥)]; Beatriz ARDIZONE JIMÉNEZ [1(¥)]; María A. JIMENEZ-SOUSA [1]; José M BELLON [2,3]; Pablo RYAN [4]; Salvador RESINO [1(*)]

(¥), Both authors contributed equally to this study; (*), Corresponding author

Current affiliations: (1) Unidad de Infección Viral e Inmunidad. Centro Nacional de Microbiología - Instituto de Salud Carlos III, Majadahonda, Spain; (2) Hospital General Universitario Gregorio Marañón, Madrid, Spain; (3) Instituto de Investigación Sanitaria Gregorio Marañón (IiSGM), Madrid, Spain; (4) Hospital Universitario Infanta Leonor (HUIL). Vallecas, Madrid, Spain.

Corresponding authors: Salvador Resino, Centro Nacional de Microbiología, Instituto de Salud Carlos III (Campus Majadahonda); Carretera Majadahonda- Pozuelo, Km 2.2; 28220 Majadahonda (Madrid), Spain. Tel: +34 918 223 266; Fax: +34 915 097 946; e-mail: sresino@isciii.es

Declarations of interest: none.

Author contributions:

Sonia Vázquez-Morón: investigation, methodology, writing – original draft

Beatriz Ardizone: investigation, methodology, writing – original draft

María A Jiménez-Sousa: investigation, methodology, writing – review and editing

José M Bellón: methodology: statistical analysis

Pablo Ryan: writing – review and editing

Salvador Resino: conceptualization, formal analysis, writing – original draft, supervision

Character count of Title: 150

Character count of Running Head: 28

Word count of Abstract: 257

Word count of Keywords: 5

Words count for main body: 4476

Count of References: 56

Count of Tables: 2

Count of Figures: 4

Count of Suppl. Data: 12

Supplemental File 5. Forest plots of sensitivity (A). specificity (B). positive LR (C). negative LR (D). diagnostic odds ratio (E). and SROC plot (F) for enzyme-linked immunosorbent assays (ELISA).

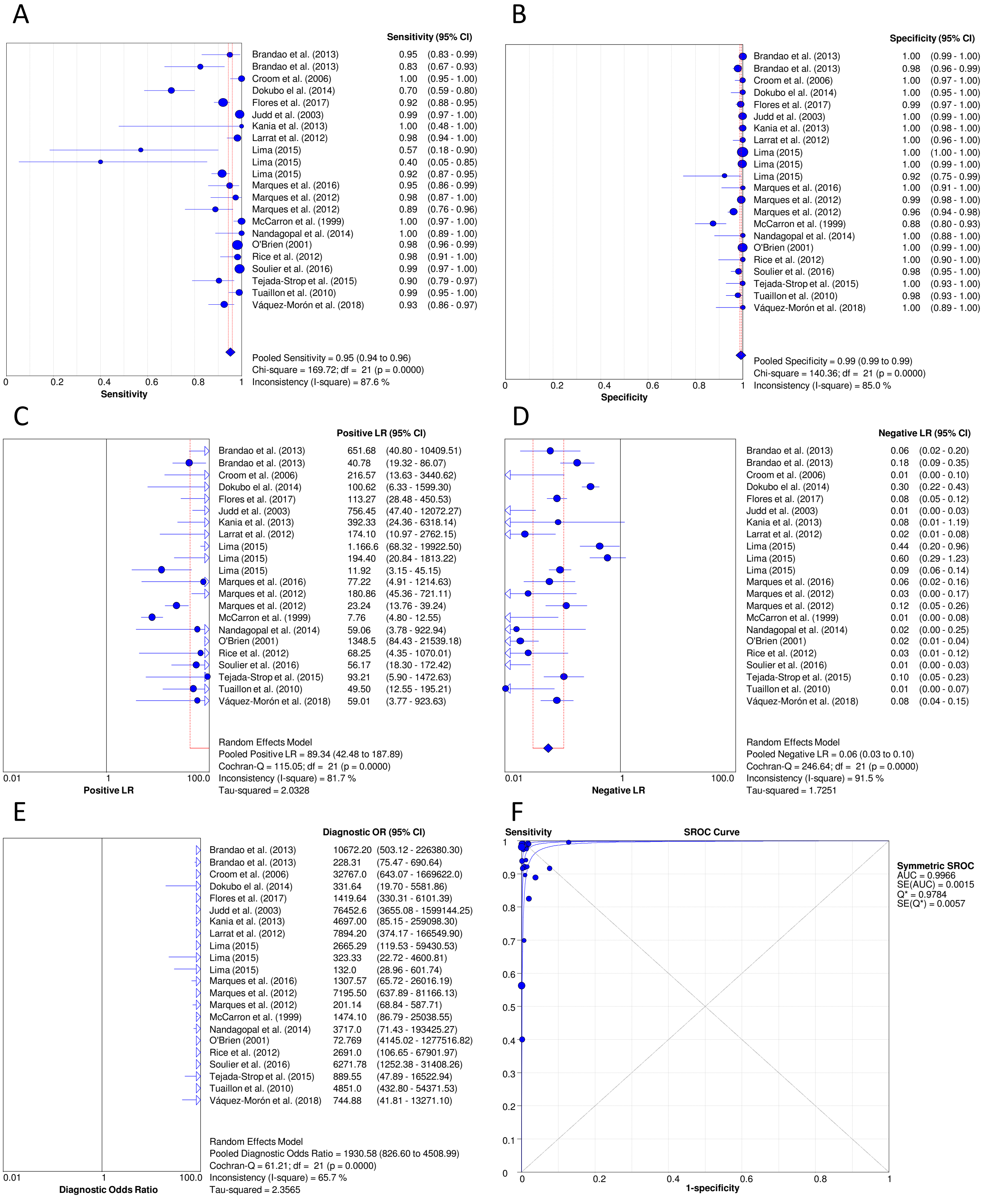

Supplement: Supplementary file 5 — Supplemental File 5 [file 41598_2019_41139_MOESM5_ESM.pdf]
