# Supplementary material for: Evaluation of the diagnostic accuracy of laboratory-based screening for hepatitis C in dried blood spot samples: A systematic review and meta-analysis
Source: Sci Rep. 2019 May 13;9:7316. doi: 10.1038/s41598-019-41139-8 (PMC6514168; doi:10.1038/s41598-019-41139-8)
Supplement: Supplementary file 8 — Supplemental File 8 [file 41598_2019_41139_MOESM8_ESM.pdf]

TITLE PAGE

Title: Evaluation of the diagnostic accuracy of laboratory-based screening for hepatitis C in dried blood spot samples: A systematic review and meta-analysis

Running head: HCV screening in DBS samples

Authors: Sonia VÁZQUEZ-MORÓN [1(¥)]; Beatriz ARDIZONE JIMÉNEZ [1(¥)]; María A. JIMENEZ-SOUSA [1]; José M BELLON [2,3]; Pablo RYAN [4]; Salvador RESINO [1(*)]

(¥), Both authors contributed equally to this study; (*), Corresponding author

Current affiliations: (1) Unidad de Infección Viral e Inmunidad. Centro Nacional de Microbiología - Instituto de Salud Carlos III, Majadahonda, Spain; (2) Hospital General Universitario Gregorio Marañón, Madrid, Spain; (3) Instituto de Investigación Sanitaria Gregorio Marañón (IiSGM), Madrid, Spain; (4) Hospital Universitario Infanta Leonor (HUIL). Vallecas, Madrid, Spain.

Corresponding authors: Salvador Resino, Centro Nacional de Microbiología, Instituto de Salud Carlos III (Campus Majadahonda); Carretera Majadahonda- Pozuelo, Km 2.2; 28220 Majadahonda (Madrid), Spain. Tel: +34 918 223 266; Fax: +34 915 097 946; e-mail: sresino@isciii.es

Declarations of interest: none.

Author contributions:

Sonia Vázquez-Morón: investigation, methodology, writing – original draft

Beatriz Ardizone: investigation, methodology, writing – original draft

María A Jiménez-Sousa: investigation, methodology, writing – review and editing

José M Bellón: methodology: statistical analysis

Pablo Ryan: writing – review and editing

Salvador Resino: conceptualization, formal analysis, writing – original draft, supervision

Character count of Title: 150

Character count of Running Head: 28

Word count of Abstract: 257

Word count of Keywords: 5

Words count for main body: 4476

Count of References: 56

Count of Tables: 2

Count of Figures: 4

Count of Suppl. Data: 12

Supplemental File 8. Meta-regression of potential sources of heterogeneity for anti-HCV antibody detection tests

Meta-Regression (Study performed after 2010)

Var	Coeff.	Std. Err.	p - value	RDOR	[95%CI]
Cte.	7.451	0.5005	0.0000	----	----
S	-0.036	0.1742	0.8362	----	----
CP_2010	2.138	0.9989	0.0415	8.49	(1.09;65.89)

Tau-squared estimate = 1.5710 (Convergence is achieved after 11 iterations)
Restricted Maximum Likelihood estimation (REML)

No. studies = 30
Filter OFF
Add 1/2 to all cells of the studies with zero

Meta-Regression (HIV coinfection)

Var	Coeff.	Std. Err.	p - value	RDOR	[95%CI]
Cte.	7.788	0.5057	0.0000	----	----
S	0.077	0.1736	0.6624	----	----
HIV	0.872	0.8345	0.3053	2.39	(0.43;13.26)

Tau-squared estimate = 1.9008 (Convergence is achieved after 9 iterations)
Restricted Maximum Likelihood estimation (REML)

No. studies = 30
Filter OFF
Add 1/2 to all cells of the studies with zero

Meta-Regression (Study performed in LMICs)

Var	Coeff.	Std. Err.	p - value	RDOR	[95%CI]
Cte.	8.523	0.4214	0.0000	----	----
S	-0.113	0.1511	0.4609	----	----
LMICs	-2.252	0.6014	0.0009	0.11	(0.03;0.36)

Tau-squared estimate = 0.6990 (Convergence is achieved after 6 iterations)
Restricted Maximum Likelihood estimation (REML)

No. studies = 30
Filter OFF
Add 1/2 to all cells of the studies with zero

Meta-Regression (Type of HCV detection test)

Var	Coeff.	Std. Err.	p - value	RDOR	[95%CI]
Cte.	7.596	0.5026	0.0000	----	----
S	0.047	0.1742	0.7886	----	----
RDT	1.654	1.0389	0.1234	5.23	(0.62;44.24)
CLA	1.200	1.2267	0.3369	3.32	(0.27;41.34)

Tau-squared estimate = 1.7506 (Convergence is achieved after 10 iterations)
Restricted Maximum Likelihood estimation (REML)

No. studies = 30
Filter OFF
Add 1/2 to all cells of the studies with zero

Meta-Regression (Anti-HCV antibody prevalence)

Var	Coeff.	Std. Err.	p - value	RDOR	[95%CI]
Cte.	7.881	1.1019	0.0000	----	----
S	0.063	0.2164	0.7731	----	----
Prevalence	0.002	0.0177	0.8923	1.00	(0.97;1.04)

Tau-squared estimate = 2.0556 (Convergence is achieved after 8 iterations)
Restricted Maximum Likelihood estimation (REML)

No. studies = 30
Filter OFF
Add 1/2 to all cells of the studies with zero

Meta-Regression (Capillary or venous DBS samples)

Var	Coeff.	Std. Err.	p - value	RDOR	[95%CI]
Cte.	8.325	0.5316	0.0000	----	----
S	-0.023	0.1891	0.9048	----	----
Capilar_DBS	-0.951	0.7769	0.2314	0.39	(0.08;1.90)

Tau-squared estimate = 1.7535 (Convergence is achieved after 8 iterations)
Restricted Maximum Likelihood estimation (REML)

No. studies = 30
Filter OFF
Add 1/2 to all cells of the studies with zero

Meta-Regression(Study Size weights)

Var	Coeff.	Std. Err.	p - value	RDOR	[95%CI]
Cte.	8.415	1.3487	0.0000	----	----
S	-0.161	0.1920	0.4100	----	----
After_2010	1.686	1.1108	0.1440	5.40	(0.54;54.38)
HIV	0.487	0.7151	0.5030	1.63	(0.37;7.20)
LMICs	-1.446	0.8733	0.1127	0.24	(0.04;1.45)
RDT	1.445	1.0599	0.1872	4.24	(0.47;38.45)
CLA	0.444	1.2302	0.7216	1.56	(0.12;20.14)
Prevalence	-0.011	0.0159	0.4877	0.99	(0.96;1.02)
Capilar_DBS	-0.752	0.6698	0.2745	0.47	(0.12;1.90)

Tau-squared estimate = 0.6703 (Convergence is achieved after 8 iterations)
Restricted Maximum Likelihood estimation (REML)

No. studies = 30
Filter OFF
Add 1/2 to all cells of the studies with zero

Abbreviations:
After_2010, study performed after 2010
Capillary DBS, capillary or venous DBS samples
CI, confidence interval
Coeff., coefficient
Cte., constant term in the equation;
HCV test, type of HCV detection test (PCR or TMA)
HIV, HIV coinfection

LMICs, study performed in LMICs
NA (---), not available
Prevalence, anti-HCV antibody prevalence
RDOR, relative diagnostic odds ratio
S, indicator of threshold.
Std. Err., standard error
Var., variables
