# Supplementary material for: Evaluation of the diagnostic accuracy of laboratory-based screening for hepatitis C in dried blood spot samples: A systematic review and meta-analysis
Source: Sci Rep. 2019 May 13;9:7316. doi: 10.1038/s41598-019-41139-8 (PMC6514168; doi:10.1038/s41598-019-41139-8)
Supplement: Supplementary file 12 — Supplemental File 12 [file 41598_2019_41139_MOESM12_ESM.pdf]

TITLE PAGE

Title: Evaluation of the diagnostic accuracy of laboratory-based screening for hepatitis C in dried blood spot samples: A systematic review and meta-analysis

Running head: HCV screening in DBS samples

Authors: Sonia VÁZQUEZ-MORÓN [1(¥)]; Beatriz ARDIZONE JIMÉNEZ [1(¥)]; María A. JIMENEZ-SOUSA [1]; José M BELLON [2,3]; Pablo RYAN [4]; Salvador RESINO [1(*)]

(¥), Both authors contributed equally to this study; (*), Corresponding author

Current affiliations: (1) Unidad de Infección Viral e Inmunidad. Centro Nacional de Microbiología - Instituto de Salud Carlos III, Majadahonda, Spain; (2) Hospital General Universitario Gregorio Marañón, Madrid, Spain; (3) Instituto de Investigación Sanitaria Gregorio Marañón (IiSGM), Madrid, Spain; (4) Hospital Universitario Infanta Leonor (HUIL). Vallecas, Madrid, Spain.

Corresponding authors: Salvador Resino, Centro Nacional de Microbiología, Instituto de Salud Carlos III (Campus Majadahonda); Carretera Majadahonda- Pozuelo, Km 2.2; 28220 Majadahonda (Madrid), Spain. Tel: +34 918 223 266; Fax: +34 915 097 946; e-mail: sresino@isciii.es

Declarations of interest: none.

Author contributions:

Sonia Vázquez-Morón: investigation, methodology, writing – original draft

Beatriz Ardizone: investigation, methodology, writing – original draft

María A Jiménez-Sousa: investigation, methodology, writing – review and editing

José M Bellón: methodology: statistical analysis

Pablo Ryan: writing – review and editing

Salvador Resino: conceptualization, formal analysis, writing – original draft, supervision

Character count of Title: 150

Character count of Running Head: 28

Word count of Abstract: 257

Word count of Keywords: 5

Words count for main body: 4476

Count of References: 56

Count of Tables: 2

Count of Figures: 4

Count of Suppl. Data: 12

Supplemental File 12. Meta-regression of potential sources of heterogeneity for HCV-RNA detection tests.

Meta-Regression(Study performed after 2010)

Var	Coeff.	Std. Err.	p - value	RDOR	[95%CI]
Cte.	7,648	0,4887	0,0000	----	----
S	-0,321	0,2923	0,3008	----	----
CP_2010	-0,772	1,2115	0,5399	0,46	(0,03;7,16)

Tau-squared estimate = 0,1218 (Convergence is achieved after 7 iterations)
Restricted Maximum Likelihood estimation (REML)

No. studies = 12
Filter OFF
Add 1/2 to all cells of the studies with zero

Meta-Regression(HIV coinfection)

Var	Coeff.	Std. Err.	p - value	RDOR	[95%CI]
Cte.	7,903	0,5027	0,0000	----	----
S	-0,330	0,2818	0,2714	----	----
HIV	-1,490	0,9604	0,1553	0,23	(0,03;1,98)

Tau-squared estimate = 0,0000 (Convergence is achieved after 1 iterations)
Restricted Maximum Likelihood estimation (REML)

No. studies = 12
Filter OFF
Add 1/2 to all cells of the studies with zero

Meta-Regression (Study performed in LMICs)

Var	Coeff.	Std. Err.	p - value	RDOR	[95%CI]
Cte.	7,701	0,5266	0,0000	----	----
S	-0,237	0,3241	0,4830	----	----
LMICs	-0,771	1,1578	0,5222	0,46	(0,03;6,35)

Tau-squared estimate = 0,0000 (Convergence is achieved after 1 iterations)
Restricted Maximum Likelihood estimation (REML)

No. studies = 12
Filter OFF
Add 1/2 to all cells of the studies with zero

Meta-Regression(Study Size weights)

Var	Coeff.	Std. Err.	p - value	RDOR	[95%CI]
Cte.	8,275	0,6304	0,0000	----	----
S	-0,261	0,2859	0,3844	----	----
PCR	-1,456	0,8653	0,1268	0,23	(0,03;1,65)

Tau-squared estimate = 0,0000 (Convergence is achieved after 1 iterations)
Restricted Maximum Likelihood estimation (REML)

No. studies = 12
Filter OFF

Add 1/2 to all cells of the studies with zero

Meta-Regression(HCV-RNA prevalence)

Var	Coeff.	Std. Err.	p - value	RDOR	[95%CI]
Cte.	7,413	2,7803	0,0258	----	----
S	-0,343	0,3252	0,3197	----	----
Prevalence	0,002	0,0430	0,9709	1,00	(0,91;1,10)

Tau-squared estimate = 0,0900 (Convergence is achieved after 8 iterations)
Restricted Maximum Likelihood estimation (REML)

No. studies = 12
Filter OFF
Add 1/2 to all cells of the studies with zero

Meta-Regression(Capillary or venous DBS samples)

Var	Coeff.	Std. Err.	p - value	RDOR	[95%CI]
Cte.	6,320	0,8672	0,0000	----	----
S	-0,467	0,2924	0,1448	----	----
Capilar_DBS	1,569	1,0021	0,1519	4,80	(0,50;46,34)

Tau-squared estimate = 0,0000 (Convergence is achieved after 1 iterations)
Restricted Maximum Likelihood estimation (REML)

No. studies = 12
Filter OFF
Add 1/2 to all cells of the studies with zero

Meta-Regression(Multivariate analysis)

Var	Coeff.	Std. Err.	p - value	RDOR	[95%CI]
Cte.	3,229	5,3307	0,5774	----	----
S	-0,348	0,4530	0,4856	----	----
CP_2010	-0,258	1,8639	0,8964	0,77	(0,00;136,52)
HIV	-3,153	2,3130	0,2445	0,04	(0,00;26,28)
LMICs	-2,632	2,5837	0,3659	0,07	(0,00;93,80)
PCR	0,947	2,5010	0,7242	2,58	(0,00;2672,47)
Prevalence	0,065	0,0739	0,4294	1,07	(0,87;1,31)
Capilar_DBS	1,612	1,6478	0,3834	5,01	(0,05;486,07)

Tau-squared estimate = 0,0000 (Convergence is achieved after 1 iterations)
Restricted Maximum Likelihood estimation (REML)

No. studies = 12
Filter OFF
Add 1/2 to all cells of the studies with zero

Abbreviations:
After_2010, study performed after 2010
Capillary DBS, capillary or venous DBS samples
CI, confidence interval
Coeff., coefficient
Cte., constant term in the equation;
HCV test, type of HCV detection test (PCR or TMA)
HIV, HIV coinfection
LMICs, study performed in LMICs
NA (---), not available

Prevalence, HCV-RNA prevalence
RDOR, relative diagnostic odds ratio
S, indicator of threshold.
Std. Err., standard error
Var., variables
